# Twelfth ISABS Conference on Forensic and Anthropologic Genetics and Mayo Clinic Lectures in Individualized Medicine, Dubrovnik, Croatia, June 22-27, 2022

**DOI:** 10.3325/cmj.2022.63.211

**Published:** 2022-06

**Authors:** Stanimir Vuk-Pavlović, Dragan Primorac

**Affiliations:** 1 International Society for Applied Biological Sciences, Zagreb, Croatia; 2 Mayo Clinic College of Medicine and Science, Rochester, MN, USA *vuk@mayo.edu*; 3 St. Catherine Specialty Hospital, Zagreb/Zabok, Croatia; 4 Eberly College of Science, Pennsylvania State University, State College, Pennsylvania, PA; 5 School of Medicine, University of Split, Split, Croatia; 6 School of Dental Medicine and Health, Josip Juraj Strossmayer University of Osijek, Osijek, Croatia; 7 School of Medicine, Josip Juraj Strossmayer University of Osijek, Osijek, Croatia; 8 Faculty of Medicine, University of Rijeka, Rijeka, Croatia; 9 Henry C. Lee College of Criminal Justice and Forensic Sciences, University of New Haven, West Haven, CT, USA; 10 Medical School REGIOMED, Coburg, Germany

With a year’s delay caused by the Sars-CoV-2 pandemic, the conference series produced by the International Society for Applied Biological Sciences (ISABS, Zagreb) is reaching its first quarter-century (1-5). During this time, it provided a forum for the crosstalk of applied genetics in forensic science, anthropology, and medicine. While the conference continues building on its tradition of interdisciplinarity, its purpose is to bring to the participants the most recent advances in its fields of focus.

The conference is preceded by the *Mayo Clinic Short Course in Epigenomics*. Here experts, mostly from the Mayo Clinic College of Medicine and Science in Rochester, Minnesota, cover the essentials of epigenomics: fundamental mechanisms underlying the regulation of gene transcription, the molecular basis of epigenetic control, epigenetic states and inheritance, epigenetics in the laboratory, and epigenetics in medicine. This is followed by the pre-conference practical *Interdisciplinary Session of the American Academy of Forensic Sciences and ISABS 2022* and *ISABS and The Ministry of Interior's Training Course: “Mystery on the Ship – Investigation of Water-Related Crime Scene.”* The *American Academy of Forensic Sciences and ISABS 2022* session focuses on standards development and progress of the Academy Standards Board, application of ancient DNA methodology in degraded forensic samples, forensic training in crime scene investigation, and aquatic death and dive accidents.

In keeping with tradition, the inaugural morning of the conference on June 22 starts with keynote lectures representing the fields covered by the conference: medicine, anthropology, and forensics. Subsequently, yielding to the urgent issues of the time, an afternoon parallel session deals with anthropology and global health in the time of crisis. It focuses on integration of global health, effects of the pandemic on global development, and data management for evidence-based response to the Sars-CoV-2 pandemic and other topics. The other parallel session covers selected topics on the role of genetic variation in disease, forensics, and anthropology.

The second day continues with anthropological aspects of global health in the time of crisis covering dietary adherence, the role of sex and geography in COVID-19 variation, etc. A parallel session covers issues of integrated high-definition omics: role of machine learning and artificial intelligence in therapy development, single-cell transcriptomics, and others. This is followed by the already traditional session where Nobel laureates address major issues of our time. This year the speakers are Aaron Ciechanover (Technion, Haifa), Sir Richard John Roberts (Northeastern University, Boston, Massachusetts), and Thomas Südhof (Stanford University, Palo Alto, California). They will address the ethical issues inherent in curing and preventing of pandemics (Ciechanover), the use of biotechnology in combatting hunger and climate change (Roberts), and biological aspects of Alzheimer’s disease (Südhof). This year, the third “Nobel Spirit” will provide the forum to the three Nobel laureates to stimulate public discussion on the role of science in solving global health issues, acute regional problems such as brain drain, demographic decline, as well as cultural and social change.

The third-day morning is dedicated to topics from anthropological genetics such as studies of ancient DNA preserved in sediments, a new development in Neanderthal genetics, and others. A part of the day is dedicated to the advancements in osteoarthritis. The second part of the day presents the talks on multi-omics of common and rare diseases as part of the program on individualized medicine.

The morning of the fourth day of the conference investigates forensic genetics cases from the one of the notorious Kaspar Hauser to the Romanov family, 9/11 victims, and more. The afternoon revisits individualized medicine through topics from epigenetics to sensitizing cancer cells to DNA damage-inducing agents, anti-tumor immunity, and others. The day ends with the evening address on creativity and artificial intelligence by Gary Kasparov, the unsurpassed former world chess champion and prominent human rights leader.

The final day of the conference returns to epigenomics in its interdisciplinary applications. Examples include the use of multi-omics in revealing therapy targets, circadian rhythm, and impact of disordered metabolism in diabetes, epigenetic signatures of psychosocial stress and trauma, and more. The conference will end with the session on the genetics of COVID-19 and pandemics such as the Black Death, as well as other topics.

This year’s conference is the first without Moses Schanfield, whom we lost last year (6). Together with us, Professor Schanfield was the founder and driving force of the conference series. We believe striving for the success of this conference and continuing the series into the future is the most fitting tribute to Professor Schanfield’s contribution to science, its dissemination, and the faithful friendship that is sorely missed.

This year again, program directors Tamás Ördög and Manfred Kayser invested their creative energy, effort, and time to compile a stellar program. Kudos should be addressed to them; shortcomings and complaints should be addressed to us.
